# Symptom load and functional status: results from the Ullensaker population study

**DOI:** 10.1186/1471-2458-12-1085

**Published:** 2012-12-18

**Authors:** Dag Bruusgaard, Hedda Tschudi-Madsen, Camilla Ihlebæk, Yusman Kamaleri, Bård Natvig

**Affiliations:** 1 Department of Community Health Institute of Health and Society, Faculty of Medicine, University of Oslo, Oslo, Norway; 2 Department of General Practice Institute of Health and Society, Faculty of Medicine, University of Oslo, P.O.Box 1130, Blindern, N-0318, Oslo, Norway; 3 Health UMB, IHA, University of Life Sciences (UMB), Aas, Norway; 4 Sintef Health Research, Oslo, Norway

**Keywords:** Functional status, Medically unexplained symptoms, Number of symptoms, Population study, Symptom reporting

## Abstract

**Background:**

There is evidence to support that the number of self-reported symptoms is a strong predictor of health outcomes. In studies examining the link between symptoms and functional status, focus has traditionally been on individual symptoms or specific groups of symptoms. We aim to identify associations between the number of self-reported symptoms and functional status.

**Methods:**

A questionnaire was sent to people in seven age groups (*N* = 3227) in Ullensaker municipality in Southern Norway. The Standardised Nordic Questionnaire and the Subjective Health Complaints Inventory were used to record 10 musculoskeletal symptoms and 13 non-musculoskeletal symptoms, respectively. Four COOP-WONCA charts were used to measure functional status.

**Results:**

We found a strong linear association between the number of self-reported symptoms and functional status. The number of symptoms explained 39.2% of the variance in functional status after adjusting for the effects of age and sex. Including individual symptoms instead of only the number of symptoms made little difference to the effect of musculoskeletal pain but affected the influence of non-muscular symptoms. Including even minor problems captured substantially more of the variance in functional status than including only serious problems.

**Conclusions:**

The strong association between the number of symptoms and functional status, irrespective of type of symptom, might indicate that the symptoms share some common characteristics. The simple act of counting symptoms may provide an approach to study the relationships between health and function in population studies and might be valuable in research on medically unexplained conditions.

## Background

Health complaints are frequently reported in the general population, but the prevalence rates vary between studies. One population study showed that about 80% of individuals experience one or more symptoms in any given month [1]. In another study, 96% of people reported at least one subjective health complaint during the previous four weeks; musculoskeletal symptoms being the most frequent complaint mentioned [2]. The size of the “symptom iceberg” in the UK has been explored in a recent article, describing the prevalence of 25 different symptoms in relation to individual characteristics and chronic conditions [3]. In this study, the mean number of symptoms experienced in the previous two weeks was 3.7. However, an estimate of only one in four individuals seeks medical attention for their symptoms [4].

Evidence supports that the number of self-reported symptoms are strongly associated with decrement in functional status. In general population based data, the number of somatic symptoms at baseline has been found to be a clinically meaningful predictor of future health status [5-7]. In clinical samples, a marked decline in functional ability has been associated with an increasing number of somatic symptoms [8,9], and patients reporting multiple somatic symptoms had worse functional ability at all time points as compared to patients with fewer symptoms [10]. In previous papers from the Ullensaker Population Study, the number of musculoskeletal pain sites explained a substantial part of the variance in functional ability and was a strong predictor of future disability pensioning [11,12]. The number of pain sites was more important than the localization of pain, and was strongly associated with the number of non-musculoskeletal symptoms [13].

Patients reporting several symptoms often represent a challenge to the health care system. In recent years, there has been increasing focus on so-called “medically unexplained symptoms” (MUS), for which there is no evident medical explanation and which are seemingly unrelated to organic disease. In a study in primary care, slightly more than half of the somatic symptoms presented were classified as physical in aetiology, more than one-third were rated as “idiopathic”, and 10% as “psychiatric” [14]. Other researchers estimate that 30–75% of physical symptoms lack a clear-cut organic cause, even after extensive diagnostic testing [8]. It has been proposed to abandon the identification of MUS, as reporting multiple *explained* symptoms and MUS have similar health outcomes. Hence, research on multisymptomatology might be increasingly important, as this term may cover the phenomenon of MUS and somatisation in future definitions and classifications [7,15-17].

In this article, we describe symptom reporting in a general population by presenting the number of symptoms reported and their association with functional status. We compare how the number of pain sites, the number of non-musculoskeletal symptoms and the total symptom load are associated with functional status measures. In addition, we explore whether knowing *which* symptoms are reported exceeds the symptom *count* in explaining the variance of functional status.

## Methods

Ullensaker is a municipality situated 40 km northeast of Oslo, the capital of Norway, and had 23,700 inhabitants in 2004. As part of a cohort study on musculoskeletal complaints starting in 1990, we sent a postal questionnaire to all inhabitants in the following birth cohorts in 2004: 1918–20, 1928–30, 1938–40, 1948–50, 1958–60, 1968–70, and 1978–80. One reminder was sent to the non-responders. The study was approved by the Regional Committee for Medical and Health Research Ethics.

Musculoskeletal symptoms were assessed using the Standardised Nordic Questionnaire (SNQ) [18]. The respondents were asked to report whether they had experienced pain or discomfort in the following 10 areas during the previous week: head, neck, shoulder, elbow, hand/wrist, upper back, lower back, hip, knee and ankle/foot (optional answers: yes/no). The regions were illustrated on a body manikin. A simple sum score, the number of pain sites (NPS) was constructed, ranging from 0 to 10.

Non-musculoskeletal symptoms were measured by a selection of 13 symptoms from the Subjective Health Complaints Inventory (SHC), which comprises 29 common health complaints [19]. The following 13 symptoms were included: palpitations, chest pain, breathing difficulties, heartburn, stomach discomfort, diarrhoea, constipation, eczema, tiredness, dizziness, anxiety, depression and sleep problems. Respondents were asked to grade the intensity of each complaint experienced over the past month on a four-point scale: not at all bothered, a little bothered, somewhat bothered and severely bothered. To correspond to “pain or discomfort” in the musculoskeletal symptom question, the answers were dichotomized into “not at all” (scored 0) vs. all other responses (scored 1), which gave an overall sum score of the number of non-musculoskeletal symptoms (NN-MS) of 0–13.

Functional status was recorded using the Norwegian version of COOP-WONCA charts [20]. In this study, we used four charts: physical fitness, feelings, daily activities and social activities. The participants were asked to rate their situation during the previous two weeks on five-point scales. Each level was illustrated pictorially, numerically and in writing. A score of 1 indicated no problem or limitation and 5 indicated maximal limitation. In this analysis, we included the four different dimensions and the sum score, which ranged from 4 to 20.

### Statistical analyses

Individuals who did not answer any symptom questions were excluded. We assumed that those who answered “yes” or “no” to at least one question about musculoskeletal symptoms meant to answer “no” to the questions left unanswered. Corresponding imputation procedures were performed for non-musculoskeletal symptoms. A full account of imputation procedures has been documented elsewhere [13]. For functional status, those who had responded to at least one of the six COOP WONCA charts were assumed to have responded “no limitations” on the remaining charts if left blank.

Descriptive statistics in the form of means and percentages, with 95% confidence intervals (CIs), were used to describe self-reported symptoms. We performed linear regression analyses, after checking for collinearity between the variables, all using the sum score of COOP-WONCA as the dependent variable. Independent variables in the different models were: Model I: NPS (the number of pain sites); Model II: NN-MS (the number of non-musculoskeletal symptoms); Model III: The total symptom load (NPS + NN-MS); Model IV: All the 10 individual musculoskeletal symptoms; Model V: All the 13 individual non-musculoskeletal symptoms; Model VI: All the individual 23 musculoskeletal and non-musculoskeletal symptoms. All variables were stratified by sex. We present the change in determination coefficient (ΔR^2^) for each component in the model, i.e. the contribution of explained variance by each variable; the unstandardised beta-value with 95% CI, and the standardised beta-value. In addition we performed analyses to obtain the measurement of fit Bayesian Information Criterion (BIC) to compare the six symptom models.

The individual symptoms were modelled individually after controlling for age, sex (Model A) and adjustment for the 22 other symptoms in addition to age and sex (Model B).

Finally, we performed linear regression analyses modelling the individual non-musculoskeletal symptoms and NN-MS in functional status/COOP-WONCA, where the cut-off for dichotomization varied: alternative 1: code 0: “not at all” + “a little” or code 1: “some” + “severe”; alternative 2: code 0: “not at all” + “a little” + “some” or code 1: “severe”.

Analyses were performed using SPSS for Windows (version 16).

## Results

The questionnaire was sent to 6105 persons, and after one reminder, 3325 individuals responded, giving a response rate of 54.4%. Individuals who had not answered any of the symptom questions were excluded (*N* = 98) as well as individuals with missing values for age, sex or any of the COOP/WONCA scales (N = 20), resulting in a final sample of 3207 individuals (52.4% of the original sample size). Of the respondents, 54.9% were women. The mean age of the sample was 47.5 (SD 14.9), women 47.2 (SD 15.0) and men 47.9 (SD14.7), both with a range from 24–86 years. An account on non-responders has been documented elsewhere [13]. Non-responders were mostly among men, and in the youngest and oldest age-groups.

**Figure 1:**
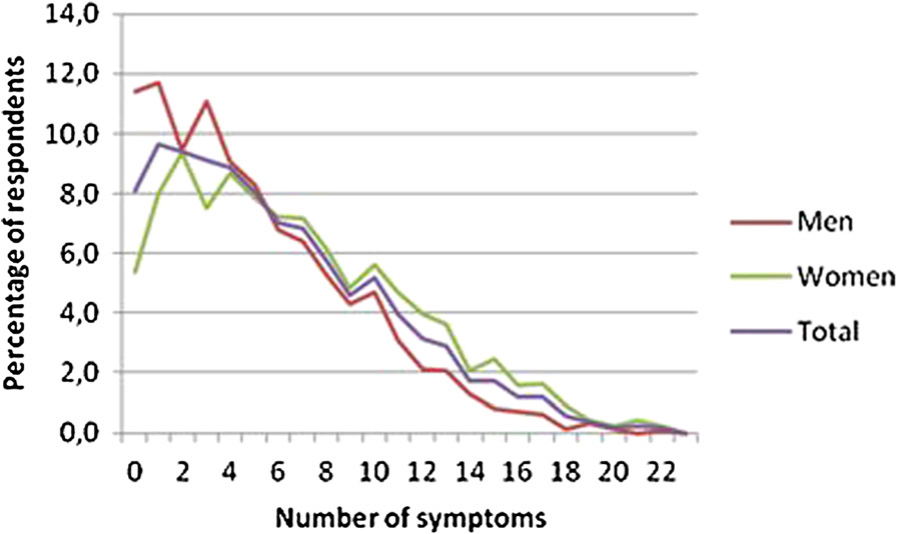
Number of symptoms reported in an adult population (percentage), stratified by sex.

A mean number of 2.3 (95% CI: 2.2-2.4) musculoskeletal symptoms was reported (women 2.8 (2.6-2.9) and men 1.8 (1.7-1.9) symptoms). The mean number of non-musculoskeletal symptoms was 3.7 (3.6-3.8) women 4.0 (3.9-4.1) and men 3.3 (3.2-3.4). The mean total symptom load (NPS + NN-MS) was 6.0 (5.9-6.2): 6.7 (6.5-7.0) in women and 5.1 (4.9-5.5) in men. No individual reported all 23 symptoms. Figure 1 shows the prevalence of the number of symptoms by sex. A total of 22.6% reported 10 symptoms or more (95% CI: 21.2–24.0), 27.8% among women (25.8–30.0), and 16% among men (14.4–18.3).

**Figure 2:**
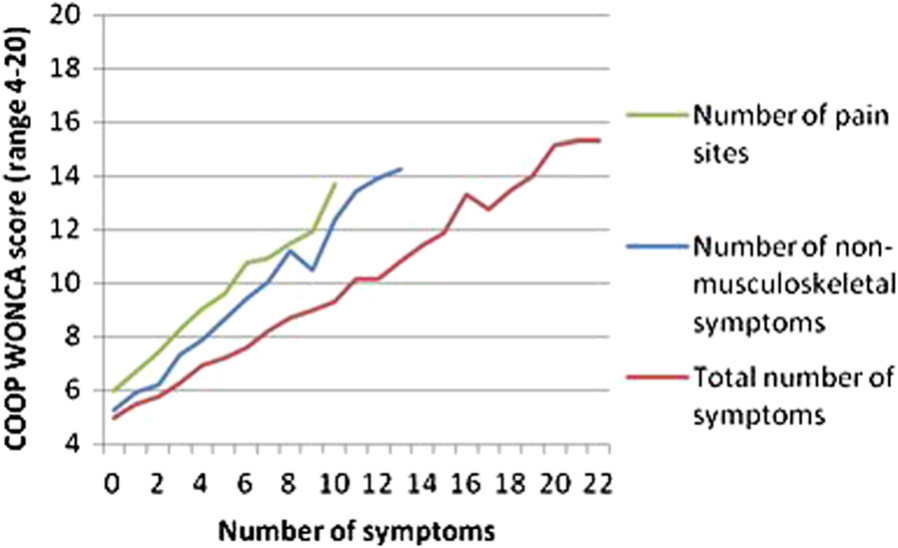
**Functional status reported in an adult population in respondents with different numbers of symptoms.** Footnote: Functional status is measured on four COOP-WONCA scales: physical fitness, feelings, daily activities, social activities, with a total range 4-20. Symptoms are grouped into Number of pain sites (0-10); Number of non-musculoskeletal symptoms (0-13) and total number of symptoms (0-23).

**Figure 3:**
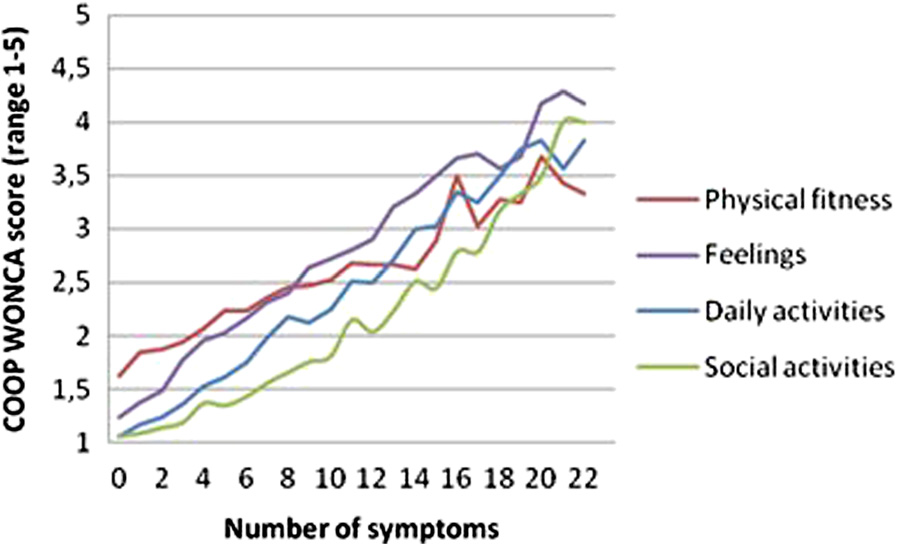
Functional status reported in an adult population as the means of four COOP/WONCA charts in respondents with different numbers of symptoms.

The number of functional problems increased linearly with an increase in NPS, NN-MS and total symptom load (NPS + NN-MS); there was no threshold or levelling out (Figure 2). Figure 3 shows how the four dimensions of functional ability increased from minor, almost no restrictions, to “substantial” restrictions as the number of symptoms increased from 0 to 22.

Table 1 shows the relationship between the number of symptoms and functional status. NPS (Model I) explained 24.9% of the variance in functional status, whereas the individual musculoskeletal symptoms (model IV) explained only slightly more (26.0%), after adjusting for the effects of age and sex. When it comes to the non-musculoskeletal symptoms, NN-MS had an explanatory power in functional status of 35.0% (Model II), whereas the individual non-musculoskeletal symptoms (Model V) had a substantially higher explanatory power (42.4%). Modelling the total symptom load (NPS + NN-MS) (Model III) rendered 39.2% explained variance in functional status, whereas all 23 individual symptoms explained 48.2% (Model VI). The explanatory power was somewhat smaller for men in all the six models (data not shown). These findings are consistent with the BIC-analyses, where the three models including individual symptoms had the lowest BIC, and hence were ranged as having the best fit to the data, compared to the models with number of symptoms (data not shown).

**Table 1:** Functional status in an adult population, as a function of individual symptoms and the number symptoms reported

		Δ**R**^**2**^	**β(95%CI)**	**Stand. Beta**	**BIC**
Model I	NPS	0.249	0.69 (0.65-0.73)	0.51	15488
Model II	NN-MS	0.350	0.68 (0.65-0.71)	0.60	14963
Model III	NPS + NN-MS	0.392	0.45(0.43-0.46)	0.64	14796
Model IV	Ind. musculoskeletal symptoms	0.260	Ind.	Ind.	15450
Model V	Ind. non-musculoskeletal symptoms	0.424	Ind.	Ind.	14540
Model VI	Ind. musculoskeletal + non-musculoskeletal symptoms	0.482	Ind.	Ind.	14224

Footnote: Functional ability is measured on four COOP-WONCA scales: physical fitness, feelings, daily activities, social activities. Symptoms are grouped in *NPS* (Number of pain sites), *NN-MS* (Number of non-musculoskeletal symptoms). ΔR^2^: Change in correlation coefficients contributed by the variable (removing effect of age); β: unstandardized regression coefficient with *CI* (Confidence interval); Stand. beta: Standardised beta values converted to the same scale for all variables. *BIC* (Bayesian Information Criterion): a measure of fit applicable for model selection, penalising for the number of variables in the model (low value indicates better fit). Ind.: each component has an individual value.

Table 2 shows the extent to which the 23 single symptoms explained the variance in functional status after age and sex adjustment (Model A). Depression and anxiety had a much higher explanatory power than the remaining symptoms, explaining 28.1% and 25.9% of the variance in functional status, respectively. The explanatory power diminished substantially or disappeared after controlling for the remaining symptoms, although some of the individual symptoms remained significant (Model B).

**Table 2:** Functional status in an adult population, as a function of separate symptoms

	**Model A**				**Model B**			
**Symptom**	Δ**R**^**2**^	**β(95%CI)**	***P*-value**	**St. β**	Δ**R**^**2**^	**β(95%CI)**	***P*-value**	**St. β**
Head	0.076	1.94 (1.72–2.16)	<0.001	0.28	0.001	0.23 (0.04–0.41)	0.017	0.033
Neck	0.088	2.01 (1.79–2.22)	<0.001	0.30	<0.001	−0.024 (−0.23–-0.18)	0.823	−0.004
Shoulders	0.089	2.03 (1.81–2.24)	<0.001	0.30	<0.001	0.075(−0.13–0.28)	0.296	0.011
Elbows	0.047	2.34 (1.99–2.68)	<0.001	0.22	0.001	0.37 (0.092–0.64)	0.009	0.034
Wrists/hands	0.069	2.23 (1.96–2.50)	<0.001	0.27	0.001	0.28 (0.053–0.51)	0.016	0.034
Upper back	0.117	2.83 (2.58–3.09)	<0.001	0.35	0.005	0.67 (0.45–0.89)	<0.001	0.082
Lower back	0.110	2.24 (2.03–2.45)	<0.001	0.33	0.007	0.65 (0.47–0.82)	<0.001	0.096
Hips	0.080	2.49 (2.21–2.77)	<0.001	0.29	0.004	0.60 (0.37–0.82)	<0.001	0.070
Knees	0.074	2.24 (1.98–2.51)	<0.001	0.27	0.005	0.65 (0.44–0.86)	<0.001	0.079
Ankles/feet	0.065	2.27 (1.98–2.55)	<0.001	0.26	0.001	0.36 (0.12–0.59)	0.03	0.041
Palpitations	0.062	1.98 (1.72–2.23)	<0.001	0.25	<0.001	0.084 (−0.12–0.29)	0.411	0.011
Chest pain	0.069	2.21(1.94–2.48)	<0.001	0.26	<0.001	0.00 (−0.22–0.23)	0.41	0.00
Breathing difficulties	0.107	2.74 (2.48–3.00)	<0.001	0.33	0.005	0.69 (0.47–0.91)	<0.001	0.083
Heartburn	0.035	1.28 (1.05–1.50)	<0.001	0.19	<0.001	−0.024 (−0.20–0.15)	0.791	−0.003
Stomach discomfort	0.079	2.15 (1.91–2.40)	<0.001	0.28	0.001	0.25 (−0.045–0.457)	0.017	0.033
Diarrhoea	0.036	1.35(1.12–1.58)	<0.001	0.19	0.001	0.19 (0.018–0.36)	0.030	0.027
Constipation	0.039	1.89 (1.58–2.20)	<0.001	0.20	0.001	0.23 (0.00–0.46)	0.050	0.025
Eczema	0.011	0.85 (0.58–1.12)	<0.001	0.10	<0.001	0.021 (−0.17–0.21)	0.212	0.003
Tiredness	0.101	2.12 (1.91–2.33)	<0.001	0.32	0.001	0.23 (0.050–0.402)	0.012	0.034
Dizziness	0.103	2.31 (2.08–2.53)	<0.001	0.32	0.001	0.28 (0.09–0.470)	0.004	0.040
Anxiety	0.259	3.94 (2.73–4.16)	<0.001	0.51	0.026	1.60 (1.37–1.83)	<0.001	0.207
Depression	0.281	3.61 (3.42–3.79)	<0.001	0.54	0.035	1.65 (1.45–1.85)	<0.001	0.245
Sleep problems	0.133	2.37 (2.18–2.58)	<0.001	0.37	0.010	0.72 (0.56–-0.89)	<0.001	0.113

Footnote: Functional status is measured on four COOP-WONCA scales: physical fitness, feelings, daily activities, social activities, and the dependent variable in all analyses is sum score of these charts. ΔR^2^: the change in R^2^ (multiple correlation coefficient) accounted for by the variable; β: unstandardised regression coefficient; CI: Confidence interval; St.β:Standardised β value. Model A: Linear regression analysis performed with individual symptoms, controlling for age and sex. Model B: Multiple regression model controlling for all other symptoms, in addition to age and sex.

We used the SNQ to ask about “pain or discomfort” without any grading of severity. Similarly, for the non-musculoskeletal symptoms, we included reports of being “a little bothered” by the symptom in question. When only those with at least “some problems” were included, the mean number of non-musculoskeletal symptoms was 1.5 (95% CI 1.4–1.5), and the explanatory power in functional ability was only slightly increased as opposed to including all levels of severity (36.1 vs. 35.0%). Including only those reporting “severe problems”, however, reduced the explanatory power in functional ability to 21.5% after adjusting for the effects of age and sex.

## Discussion

A substantial proportion of the population reports a variety of symptoms. We have documented a strong linear association between number of self-reported symptoms and functional status. Including the individual symptoms instead of the number of symptoms made little difference to the influence of musculoskeletal pain, but affected the influence of non-musculoskeletal symptoms more. The influence of single symptoms in explaining the variance in functional ability decreased after controlling for the total burden of other symptoms. Including even minor problems captured substantially more of the association with function compared to including only serious problems.

## Methods

Even though we used media to motivate participation, the response rate was modest. The Ullensaker Population Study has used the SNQ to measure musculoskeletal symptoms since the start in 1990. When we decided to also include non-musculoskeletal symptoms, we chose to use a modified version of a validated instrument, the SHC. Hence, there is a temporal mismatch between the two instruments, the one-week window in the SNQ and the 30-day window in the SHC. Consequently, our results are not a consistent measurement of the total symptom count for a defined period. The two-week window in the WONCA charts adds to the mismatch. Because our interest is mainly the association between symptoms and other variables, and because the associations were so strong, our conclusions should still be valid despite the low response rate and methodological shortcomings.

The results from this study might partly reflect reporting behaviour. Some persons might tend to report any discomfort, whereas others do not mention minor discomfort. However, we have previously shown that the number of pain sites strongly predicts future disability pensioning [12]. Non-responders in this study were mostly among groups reporting the least symptoms (men and youngest and oldest age groups), indicating that our findings might overestimate symptom prevalences.

## Results

Although single symptoms are important in the clinical setting, the number of symptoms seems to be an important dimension in population studies. This phenomenon has attracted increasing attention in recent years [3,11,12,21]. However, different studies have used different methods including the recording of symptoms, symptom definitions and time windows.

There is reason to believe that patients do not report in the same way during a clinical consultation and that health professionals do not know the full spectrum of symptoms experienced by their patients. We included minor problems, which are seldom presented in medical encounters, as well as more serious problems, which would be presented in medical encounters; hence, our study presents the whole iceberg of symptoms.

It seems that the total burden of symptoms is an important dimension of functional ability. We are the first to show that functional ability decreases linearly and does not level out throughout the whole range of symptoms, without distinguishing between musculoskeletal or non-musculoskeletal symptoms, or between mental and somatic symptoms. Patients with a number of controversial diagnoses, such as chronic fatigue syndrome, irritable bowel syndrome or fibromyalgia, or patients with the overriding concept of medically unexplained symptoms, all report many symptoms and were probably included among those reporting the most symptoms in our study.

A UK-wide population study of a similar size and including a similar number of symptoms (25 vs. the 23 in this study), found a mean of 3.7 symptoms among respondents [3], a substantially lower mean than our 6.0. Although methodological differences might explain part of the difference, Norwegians regularly report many symptoms in health examination studies. A European study of chronic pain showed that Norwegians were on top with a prevalence of 30%, compared to 13% of the UK responders [22].

We found that the 23 individual symptoms explained almost half of the variance in functional status, which must be considered a substantial contribution. The explanatory powers of the models in Table 1, as measured by ΔR ^2^, are not directly comparable. The models including the individual symptoms have a higher number of variables which in itself renders higher R^2^. However, the findings were also supported by use of another measure of fit, BIC, which showed the same ranking of the models (Table 1).

Although the number of symptoms does not completely capture the explanatory power of individual symptoms, it seems that the number may be an acceptable proxy and provide substantial information in a population setting. Including the number of symptoms instead of individual symptoms may provide statistical advantage in smaller population sizes, such as in clinical settings.

All individual symptoms contributed to explaining the variance in functional status, but only a few remained significant after adjusting for the total symptom load. This is not surprising, since the individual symptoms are interrelated.

We found that anxiety and depression were symptoms that had substantially higher explanatory power in functional status than other symptoms. This effect was reduced, but still statistically significant, after controlling for report of remaining symptoms. Other studies have found a strong association between number of symptoms and psychiatric morbidity [1,8,23,24]. One study has documented that the number of somatic symptoms and anxiety/depression independently account for impairment in health related quality of life [25]. However, we propose that a differentiation between mental and somatic symptoms should be avoided in future generic epidemiological studies.

In this paper we have looked at associations between symptoms and functional status, not causal relationships. Exploratory analyses indicate that socioeconomic status (measured as marrital status, educational level and employment status in our study) contribute to approximately 16% of the variance in functional status if included in the models in Table 1.

The strong association between the number of symptoms and functional status might indicate that the symptoms share some common characteristics, regardless of whether these are mental or somatic. There is a need for a common instrument to explore the phenomenon of symptom reporting in the population to make a comparison of studies easier.

## Conclusion

The simple act of counting symptoms provides an approach for population-based studies and might even be valuable in research on medically unexplained symptoms. Whether the method of counting symptoms will have consequences for future clinical work and in what manner remains to be seen.

## Abbreviations

MUS: Medically unexplained symptoms; SNQ: Standardised Nordic Questionnaire; SHC: Subjective Health Complaints Inventory; NPS: Number of pain sites; NN-MS: Number of non-musculoskeletal symptoms.

## Competing interests

The authors declare that there are no competing interests.

## Authors’ contributions

DB designed the study and drafted the manuscript. CI, YK and BN contributed to conception and design, acquisition of data, and revised manuscript for intellectual content. HTM helped perform the statistical analysis and interpretation of data, and helped draft the manuscript. All authors read and approved of the final manuscript.

## Pre-publication history

The pre-publication history for this paper can be accessed here:

http://www.biomedcentral.com/1471-2458/12/1085/prepub
